# Ion Torrent and lllumina, two complementary RNA-seq platforms for constructing the holm oak (*Quercus ilex*) transcriptome

**DOI:** 10.1371/journal.pone.0210356

**Published:** 2019-01-16

**Authors:** Victor M. Guerrero-Sanchez, Ana M. Maldonado-Alconada, Francisco Amil-Ruiz, Andrea Verardi, Jesús V. Jorrín-Novo, María-Dolores Rey

**Affiliations:** 1 Agroforestry and Plant Biochemistry and Proteomics Research Group, Department of Biochemistry and Molecular Biology, University of Cordoba, Cordoba, Spain; 2 Bioinformatics Unit, Servicio Central de Apoyo a la Investigación (SCAI), University of Cordoba, Cordoba, Spain; 3 Thermo Fisher Scientific, Alcobendas, Madrid, Spain; Jawaharlal Nehru University, INDIA

## Abstract

Transcriptome analysis is widely used in plant biology research to explore gene expression across a large variety of biological contexts such as those related to environmental stress and plant-pathogen interaction. Currently, next generation sequencing platforms are used to obtain a high amount of raw data to build the transcriptome of any plant. Here, we compare Illumina and Ion Torrent sequencing platforms for the construction and analysis of the holm oak (*Quercus ilex*) transcriptome. Genomic analysis of this forest tree species is a major challenge considering its recalcitrant character and the absence of previous molecular studies. In this study, *Quercus ilex* raw sequencing reads were obtained from Illumina and Ion Torrent and assembled by three different algorithms, MIRA, RAY and TRINITY. A hybrid transcriptome combining both sequencing technologies was also obtained in this study. The RAY-hybrid assembly generated the most complete transcriptome (1,116 complete sequences of which 1,085 were single copy) with a E90N50 of 1,122 bp. The MIRA-Illumina and TRINITY-Ion Torrent assemblies annotated the highest number of total transcripts (62,628 and 74,058 respectively). MIRA-Ion Torrent showed the highest number of shared sequences (84.8%) with the oak transcriptome. All the assembled transcripts from the hybrid transcriptome were annotated with gene ontology grouping them in terms of biological processes, molecular functions and cellular components. In addition, an in silico proteomic analysis was carried out using the translated assemblies as databases. Those from Ion Torrent showed more proteins compared to the Illumina and hybrid assemblies. This new generated transcriptome represents a valuable tool to conduct differential gene expression studies in response to biotic and abiotic stresses and to assist and validate the ongoing *Q*. *ilex* whole genome sequencing.

## Introduction

Holm oak (*Quercus ilex* L.) forms natural forests or “dehesa” ecosystems, playing an important role from an environmental and socio-economic point of view [1]. Holm oak, as with other forest tree species, can be defined as an orphan and recalcitrant experimental system, whose study at the molecular and genomic level represents a challenge. To date, partial studies using classical biochemical and proteomics approaches have shed some light on different aspects of *Q*. *ilex* biology such as natural variation, seed germination, seedling growth, physiology and, biotic and abiotic stress-responses [2–10].

The holm oak genome has not yet been sequenced, however, transcriptome analysis, using RNA-sequencing (RNA-Seq), offers an alternative technology now widely used to identify and characterize gene sequences [11–12]. In order to generate transcriptomes, a set of read sequences are obtained first by next generation sequencing (NGS) technologies. Of these, Illumina is the most commonly used. However, an alternative technology is provided by Ion Torrent instruments. The raw read data obtained using both platforms differ in some parameters such as fragment length, probability of base substitutions or insertion/deletion alterations in homopolymeric regions [13]. Once generated, these reads must be *de novo* assembled to produce a transcriptome. Several *de novo* transcriptome assemblers are currently available [14–15] that, combined with user-tunable parameters, enable the generation of a large figure of candidate assemblies for a single data set.

Recent studies have shown that the evaluation of *de novo* transcriptome assemblies remains a challenge [12], [16], and there is not a universal accepted optimal assembler identified for *de novo* generation.

Recently, a *de novo* transcriptome assembly of *Q*. *ilex* was published using an Illumina Hiseq 2500 platform [17–18]. Initially, 31,973 total sequences were annotated using the Blast2Go software [17] and later, the total number of transcripts was increased to 62,628 total sequences using the Sma3s v2 software [18]. To improve the amount of annotated sequences, in this work, we compare the resulting assembled sequences from two sequencing platforms, the new Ion Torrent reads against the Illumina transcriptome previously described by our group [17–18]. In addition, a hybrid transcriptome obtained from Illumina and Ion Torrent combined reads is discussed. It should be noted that the data obtained from each sequencing platform depends on the organism on study. Every species has a different number of genes which requires a tailored sequence yield for an effective transcriptome [19]. Moreover, a comparison of three assemblers (MIRA, TRINITY and RAY), each using different algorithms, for the construction of a new *de novo* transcriptome of holm oak is carried out in each platform and then compared to each other. The assemblies to provide a transcriptome are highly variable in the contigs and scaffold lengths, and in the total assembly size [16], [20].

## Materials and methods

### Plant material

Mature acorns from holm oak (*Quercus ilex* L. subsp. *ballota* [Desf.] Samp.) were collected from a tree located in Aldea de Cuenca (province of Cordoba, Andalusia, Spain). Acorns were germinated, and seedlings grown in a chamber under controlled conditions previously described in [17]. Germinated embryos, leaves and roots from 6-months plantlets were collected and individually frozen in liquid nitrogen. The plant material used for RNA sequencing experiments consisted of a pool generated by mixing equal amounts of homogenized tissue from acorn embryos, leaves and roots.

### RNA extraction

Total RNA was extracted from the frozen homogenized pool tissue following the procedure previously reported by Guerrero-Sanchez et al. [17]. A total of 50 mg pooled fresh tissue was used following the protocol previously described by Echevarría-Zomeño et al. [3]. Contaminating genomic DNA was removed by DNase I treatment (Ambion, Austin, TX). Total RNA was quantified spectrophotometrically (DU 228800 Spectrophotometer, Beckman Coulter, TrayCell Hellma GmbH & Co. KG), and the integrity of the isolated RNA was assessed using a 2100 Bioanalyzer (Agilent Technologies, Palo Alto, Calif.). Only high-quality RNAs with RIN values > 8 and A_260_:A_280_ ratios near 2.0 were used for subsequent experiments.

### RNA-Seq Library Construction, Illumina sequencing and *de novo* assembly

The holm oak Illumina transcriptome was previously described in [17–18]. Briefly, the library construction of cDNA molecules for Illumina sequencing was carried out by Illumina TruSeq Stranded mRNA library preparation kit using 2 μg of total RNA. The cDNA was synthesized and sequenced in the Illumina Hiseq 2500 platform and three different assemblers (TRINITY 2.5.1 [21], RAY 2.3.1 [22] and MIRA 4.9.6 [23] algorithms) were employed to *de novo* assemble the *Q*. *ilex* transcriptome. Both the length and distribution of Illumina reads are shown in S1 Fig

### RNA-Seq Library Construction, Ion Torrent sequencing and *de novo* assembly

The cDNA library was built using the Ion Total RNA-Seq Kit v2 for whole transcriptome libraries (Life Technologies Corporation, California, USA), using an aliquot from the same RNA used for Illumina. Thus, 10 ng and 50 ng of total RNA were employed to generate in parallel two cDNA libraries that were loaded by an Ion Chef System in two Ion 540 sequencing chips and then, further sequenced by an Ion S5 System. Raw reads with length up to 372 nucleotides (mean of 112 nucleotides) from each sequencing chip were processed to filter out poor quality sequences (Cutadapt version 1.9 (-m 100) and BBDuk version 35.43 (qtrim = rt trimq = 20)). Sequencing adapters were first clipped, and low-quality bases (with phred score below a threshold) were trimmed in raw sequences. A phred score value was selected as thresholds (20) and reads shorter than 100 nucleotides were filtered out. Both the length and distribution of Ion Torrent reads are shown in S1 Fig The processed reads were assembled into contigs using the same assemblers (TRINITY version 2.5.1, RAY version 2.3.1 and MIRA version 4.9.6) used to obtain the Illumina transcriptome described in the previous section, but the parameterizations were: TRINITY chosen parameters: “—max_memory 1000G —CPU 20 --SS_lib_type F --bflyCalculateCPU—normalize_max_read_cov 20”, with “—KMER_SIZE 25”, also with and without “—min_kmer_cov 2”. RAY chosen parameters were: “-n 22”, and for “-k 31”. MIRA chosen parameters were: “job = denovo,est,accurate; COMMON_SETTINGS -GENERAL:number_of_threads = 12 -KMERSTATISTICS:lossless_digital_normalisation = yes; IONTOR_SETTINGS -ALIGN:min_relative_score = 70 -ASSEMBLY:minimum_read_length = 100; -CLIPPING:quality_clip = no -CLIPPING:qc_minimum_quality = 15 -CLIPPING:qc_window_length = 20; -CLIPPING:clip_polyat = yes -CLIPPING:cp_min_sequence_len = 12; technology = iontor”.

As with the Illumina transcriptome, the assembly calculations were run in the Computations Cluster of CICA (Centro de Información Científica de Andalucía, Spain) (https://www.cica.es/servicios/supercomputacion/), the supercomputing and bioinnovation center service of the University of Malaga (Spain) (http://www.scbi.uma.es/site/), and the supercomputing facilities of the Research, Technological Innovation and Supercomputing Center of Extremadura, Spain (http://www.cenits.es/).

### Development of a hybrid transcriptome

A *de novo* hybrid transcriptome was also built using both Ion Torrent single-end and Illumina paired-end reads. Considering tested computational requirements and performance in the tests carried out in the *de novo* hybrid transcriptome, the RAY assembler was selected to carry out the hybrid assembly using raw data from both sequencing platforms, with the parameter *k-mer* = 31. In addition, we built a partial hybrid transcriptome using a random-selection of half of the Illumina reads, and half of the Ion Torrent reads, with the aim of checking if the good quality of the hybrid transcriptome was only due to the read depth of using two sequencing platforms. The partial hybrid transcriptome, using randomly-selected halves of the Illumina and Ion Torrent reads is designed as partial hybrid transcriptome in the manuscript.

### Assembly quality and completeness evaluation

The evaluation of the structure of the generated assemblies from both sequencing platforms was performed using QUAST (version 5.0.0). The QUAST software [24] generates an overview of the sizes distribution (including largest contig, total length, N50, L50, N75, L75, and GC (%)) of the contigs contained in every *de novo* transcriptome. Moreover, a re-alignment of all the assemblies was carried out to obtain more transcriptome-specific metrics such as E90N50 transcript contig length, DETONATE score values, number of alignable reads and alignments in total using DETONATE (version 1.11) [25] in each assembly. DETONATE (DE novo TranscriptOme rNa-seq Assembly with or without the Truth Evaluation) evaluates *de novo* transcriptome assemblies by two component packages, RSEM-EVAL and REF-Eval, providing a rigorous computational assessment of the quality of a transcriptome assembly and the best assembly is the one with the highest DETONATE score (http://deweylab.biostat.wisc.edu/detonate/). The assembly quality for Illumina assemblies was previously reported in [17], so it was omitted.

The completeness of all the transcriptomes obtained from Illumina, Ion Torrent and hybrid transcriptome data was evaluated using Benchmarking Universal Single-Copy Orthologs (BUSCO) following the BUSCO v3 user guide (version 3.0.2) using as commands “Python run_BUSCO.py–i sequence_file–o output_name–l lineage–m tran” and “Python generate_plot.py–wd working directory” [26–27]. A complete annotation of the *Q*. *ilex* transcriptome assembled from both Ion Torrent and hybrid transcriptome data (both whole and partial hybrid transcriptomes) was carried out by using the Sma3s v2 annotator [28–29].

### *De novo* transcriptome alignment with *Quercus robur* and *Quercus petrea* transcriptomes

All the assemblies obtained in this work were aligned with the most complete and annotated transcriptome sequences of *Q*. *robur* and *Q*. *petrea* (http://www.oakgenome.fr) (OCV4 transcriptome version), both species being phylogenetically close to holm oak. *Quercus robur* and *Q*. *petrea* transcriptomes are designated as oak transcriptomes in the manuscript [30]. The alignment software used was blastN [31] with an e-value cutoff of 10^−30^. Alignment blast outputs were graphically and statistically analyzed using R 3.5.0 and RStudio 1.1.447 [32–33].

### Identification of proteins from translated assemblies used as databases

A protein identification using a holm oak peptide spectra sample previously described in [18] was used in this study. A 6-frame translation for each sequence, in all the transcriptomes generated, was performed using EMBOSS (version 6.6.0) [34], filtering and keeping peptides longer than 50 amino acids using the R package Biostrings (version 2.48.0) [32–33], [35]. The resulting FASTA files were used individually as a custom holm oak protein database for the protein identification. Spectra were processed using the SEQUEST algorithm available in Proteome Discoverer 2.1 (Thermo-Scientific, Massachusetts, USA). The following settings were used as previously described in Romero-Rodríguez et al. [10]: precursor mass tolerance was set to 10 ppm and fragment ion mass tolerance to 0.8 Da. Only charge states +2 or greater were used. Identification confidence was set to a 5% FDR, the variable modifications were set to: oxidation of methionine, and the fixed modifications were set to carbamidomethyl cysteine formation. A maximum of two missed cleavages were set for all searches.

## Results

### Sequencing platforms and *de novo* assembly structure analysis

To compare the transcriptome features obtained from two different sequencing platforms, equal quantities of total RNA from three tissues, acorn embryos, leaves and roots of holm oak were mixed and used to construct a cDNA library for sequencing based on the Illumina HiSeq2500 and Ion Torrent S5 platforms. A total of 55,275,472 Illumina paired-end reads and 55,161,453 (10 ng of total RNA) and 84,364,256 (50 ng of total RNA) Ion Torrent single-end reads were generated in this study. The raw reads were preprocessed to eliminate primer/adaptor contamination and low-quality section of reads, generating a total of 50,870,724 and 46,334,832 (both RNA concentrations were preprocessed together) clean raw data in Illumina and Ion Torrent, respectively. In each sequencing platform used, the assembly was performed by three different assemblers (MIRA, RAY and TRINITY) and compared to each other (Table 1). However, the hybrid assemblies were built using only the RAY assembler, since TRINITY does not allow the construction of a hybrid assembly and MIRA requires many computational resources when a hybrid assembly is built (Table 1).

**Table 1:** Summary of the structure of the holm oak assembly.

Assembly structure								
	Illumina*			Ion Torrent			Hybrid	Hybrid half
	MIRA	RAY	TRINITY	MIRA	RAY	TRINITY	RAY	
# contigs (≥ 0 bp)	169449	107487	77159	710041	107497	303541	132720	104640
# contigs (≥500 bp)	43014	20495	8803	22879	18551	118726	26670	21041
# contigs (≥ 1000 bp)	15445	8773	696	5017	5233	49190	13779	11715
# contigs (≥ 5000 bp)	155	73	1	1	4	118	185	173
# contigs (≥ 10000 bp)	2	3	0	0	0	2	9	7
Largest contig	11254	12220	5916	5273	5533	11940	15329	15043
Total length (≥ 0 bp)	83639406	41292773	26286544	145717222	35361128	185129754	56442863	45257060
Total length (≥ 1000 bp)	27409911	14778197	904440	7040671	7467041	79149878	25612168	22023591
Total length (≥ 5000 bp)	941227	471829	5916	5273	21202	710782	1206376	1107152
Total length (≥ 10000 bp)	21731	34168	0	0	0	22544	112633	82952
N50	1211	1260	661	839	930	1206	1558	1630
E90 number of transcripts	127958	65285	64150	584912	66454	224685	71023	63138
E90N50	673	806	361	215	579	946	1122	1188
Score	-2334943804	-3400761031	-6756877372	-6686768444	-5727910347	-4259931488	-7602101330	-1455877920
Number of alignable reads	48681788	39297987	9787481	35784571	27414374	42141854	82372290	22202091
Number of alignments in total	169413628	48341674	15563083	267150454	34964535	631869894	109250751	29749610
N75	742	827	563	628	685	797	972	1042
L50	11473	5863	3428	7718	6149	35219	7174	5731
L75	23813	11529	5931	14324	11404	67779	14209	11200
GC (%)	41,69	42,47	39,14	42,30	42,76	42,04	41,44	42,07

*Data from the Illumina platform were previously published in [17].

The assembly structure analysis was carried out by the QUAST software, which provided an overview of the number of contigs longer than a concrete base pairs length (from ≥ 0 bp to ≥ 10,000 bp) (Table 1), together with other statistical parameters such as N50, N75, L50, L75 and % GC (Table 1). Moreover, the assembly structure analysis was complemented with other transcriptome-specific metrics (E90N50, overall score values, length of alignable reads and number of alignments in total) obtained by using the DETONATE software (Table 1). In the case of contigs ≥ 10,000 bp, both the Illumina and hybrid assemblies resulted in a low number of contigs using MIRA (Illumina, 2 contigs), RAY (Illumina, 3 contigs), TRINITY (Ion Torrent, 2 contigs) and RAY (hybrid assembly, 9 contigs and partial hybrid assembly, 7 contigs). The number of contigs between 1,000 and ≥ 5,000 bp was much higher in the TRINITY-Ion Torrent assembly (118 and 49,190 contigs, respectively) and the MIRA-Illumina assembly (155 and 15,445 contigs, respectively) than when the other assemblers were used (Table 1). The highest number of contigs in holm oak was observed in those contigs between 0 bp and ≥ 500 bp. Both the MIRA-Illumina assembly (169,449 and 43,014 contigs, respectively) and the MIRA-Ion Torrent assembly (710,041 and 22,879, respectively) showed the highest number of these contigs (Table 1). The largest contig was constructed by RAY using the hybrid assembly reads (15,329 bp) (Table 1). However, from Illumina reads, the largest contig was obtained by RAY (12,220 bp), while from Ion Torrent reads, the largest contig was obtained by TRINITY (11,940 bp) (Table 1). The maximum total length of annotated sequences (≥ 10,000 bp) was yielded in the RAY hybrid assembly (112,633 bp). Neither the TRINITY (Illumina) assembly nor MIRA and RAY (Ion torrent) assemblies showed sequence lengths higher than 10,000 bp. For ≥ 5,000 bp total lengths of annotated sequences, RAY hybrid assembly showed more annotated sequences (1,206,376 bp) and for ≥ 1,000 bp total length of annotated sequences, MIRA-Illumina (27,409,911 bp) and TRINITY-Ion Torrent (79,149,878 bp) assemblies showed more annotated sequences than in the remaining assemblies (Table 1). For annotated sequences of a total length of ≥ 0 bp, MIRA-Illumina (83,639,406 bp) and TRINITY-Ion Torrent (185,129,754 bp) assemblies showed the highest number of annotated sequences in holm oak (Table 1). The contig N50, in the Ion Torrent platform, was higher in TRINITY (1,206 bp) than in MIRA (930 bp) and RAY (839 bp) and, in the Illumina platform, was practically equal using MIRA (1,260 bp) and RAY (1,211 bp) (Table 1). The N50 value was 1,558 bp in the hybrid transcriptome and 1630 bp in the partial hybrid transcriptome (Table 1). The GC % content was quite similar in all the assemblers (Table 1). In addition, we analysed the transcriptome-specific measurement E90N50 because it is a preferable parameter over the original N50 when evaluating transcriptome assemblies [36]. Both hybrid assemblies (1,122 bp in the hybrid transcriptome and 1,188 bp in the partial hybrid transcriptome) showed the highest E90N50 values in this study, followed by RAY-Illumina (806 bp) and TRINITY-Ion Torrent (946 bp) (Table 1). The best DETONATE score values were observed in the partial hybrid transcriptome (-1,455,877,920 bp) and MIRA-Illumina (-2,334,943,804 bp) (Table 1). With regard to the number of alignable reads and total alignments, both the hybrid assembly (82,372,290) and TRINITY-Ion Torrent (109,250,751) were higher than the rest of assemblies, respectively (Table 1).

The efficiency of the use of resources of each assembler should be considered in a transcriptome analysis; therefore we monitored this for MIRA, TRINITY and RAY in the Illumina, Ion Torrent and hybrid transcriptomes. The MIRA-Illumina assembler used a higher amount of resources, more than 40 central processing units (CPUs) in some points and a mean of 174.80 GB of RAM memory (S2b Fig). The TRINITY-Illumina assembler used many resources during the first minutes of the assembly process, but later, only one core and a mean of 0.55 GB of RAM were used for the final process of the assembly (S2c Fig). However, this assembler created an immense amount of files. Finally, the RAY-Illumina assembler was the most efficient in the use of resources from the Illumina reads, considering that a mean of 10.73 GB of RAM was used (S2a Fig). In addition, RAY did not generate weighty temporary files, and only used a few MB necessary for the assembly and the logs of the process. The MIRA-Ion Torrent assembler used a mean of 95.85 GB of RAM memory (S3b Fig). The TRINITY-Ion Torrent assembler used, as TRINITY-Illumina, many resources at the beginning of the assembly process, and a mean of 0.90 GB of RAM (S3c Fig). From the Ion Torrent reads, the RAY assembler was also the most convenient in terms of computational resources compared to the other assemblers analyzed in this study (15.61 GB of RAM) (S3a Fig). Regarding the RAY-hybrid assemblers, a mean of 13.55 GB of RAM was used in the hybrid transcriptome assembly and a mean of 15.62 GB of RAM was used in the partial hybrid transcriptome assembly (S4a and S4b Fig).

### *Quercus ilex de novo* transcriptome alignment with *Q*. *robur* and *Q*. *petrea* transcriptomes

**Fig 1:**
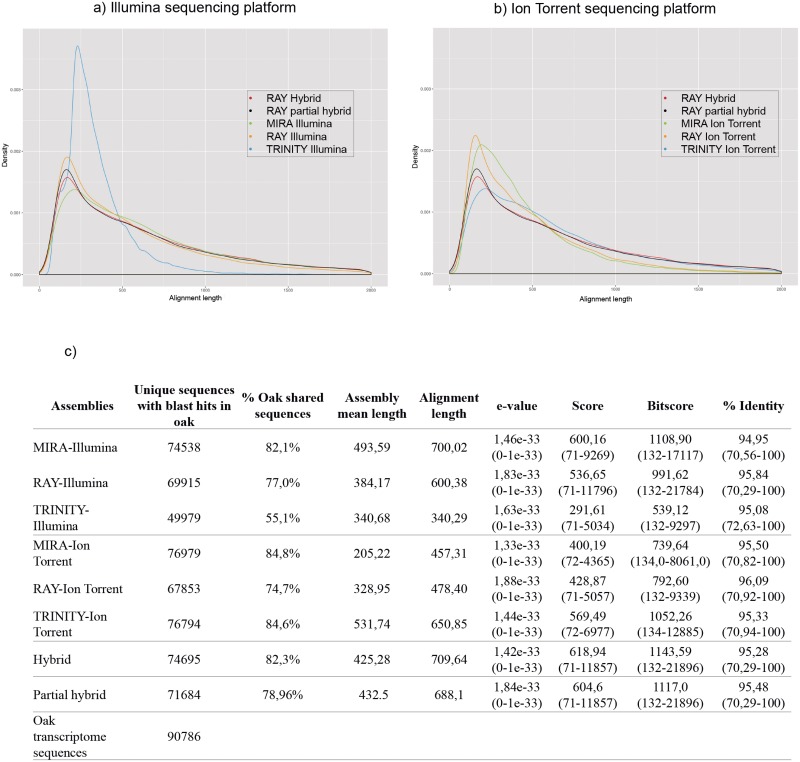
Alignment between *Q*. *robur* and *Q*. *petrea* transcriptomes (oak transcriptome) and *Q*. *ilex* (holm oak) transcriptome using MIRA, RAY, TRINITY and RAY hybrid assemblies from Illumina (**a**) and Ion Torrent (**b**) reads. Distribution of percent sequence identity between oak and *Q*. *ilex* (MIRA, RAY, TRINITY, RAY hybrids) transcriptomes (**c**).

An alignment between the holm oak transcriptome and the *Q*. *robur* and *Q*. *petrea* transcriptomes was carried out through a local alignment using blastN with the oak transcriptome as a database and the new assemblies obtained in this work as queries. As a result, a density graph was generated with the length of the oak transcriptome and the *Q*. *ilex* transcriptome built by all the assemblers used (Fig 1). From Illumina reads, MIRA built the best assembly (Fig 1a), as previously described [17]. From Ion Torrent, TRINITY-Ion Torrent built the best assembly (Fig 1b). The oak transcriptome and *Q*. *ilex* (MIRA-Illumina) transcriptome showed 82.1% of shared sequences (Fig 1c), followed by RAY-Illumina (77.0%) and TRINITY-Illumina (55.1%) (Fig 1c). From Ion Torrent reads, MIRA built the best assembly with 84.8% of shared sequences between oak and *Q*. *ilex* transcriptomes, followed by TRINITY (84.6%) and RAY (74.7%) (Fig 1c). The *Q*. *ilex* hybrid transcriptome and the *Q*. *ilex* partial hybrid transcriptome showed 82.3% and 78.9% of shared sequences with oak transcriptome, respectively (Fig 1c). The distribution of percentage sequence identity between oak and *Q*. *ilex* (MIRA, RAY and TRINITY) transcriptomes from Illumina, Ion Torrent and hybrid reads was also analyzed (Fig 1c). The highest percentage of identity was observed in the RAY-Ion Torrent assembly (96.1%), followed by the RAY-Illumina assembly (95.8%) (Fig 1c).

### Transcriptome completeness evaluation

**Fig 2:**
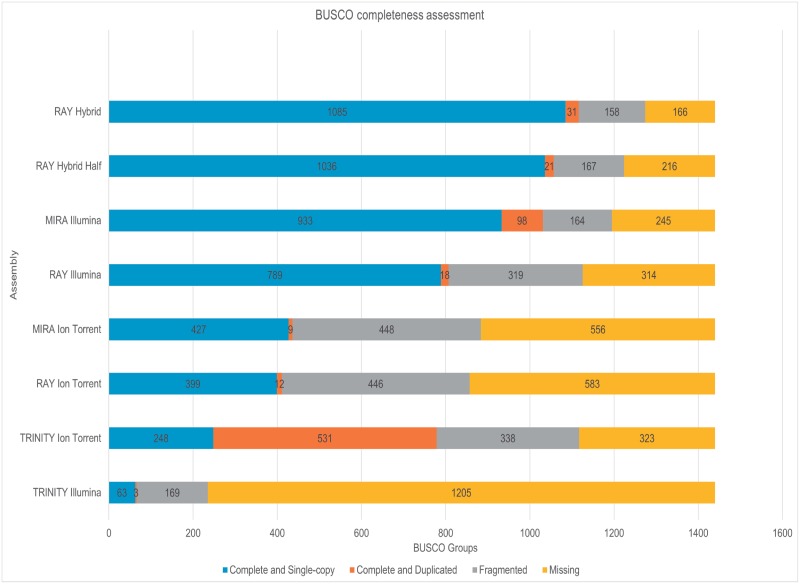
Results of BUSCO analysis of the holm oak transcriptome. All the transcriptomes are organized depending on their completeness: RAY-hybrid assembly, RAY-partial hybrid assembly, MIRA-Illumina assembly, RAY-Illumina assembly, MIRA-Ion Torrent assembly; RAY-Ion Torrent assembly; TRINITY-Ion Torrent assembly; and TRINITY-Illumina assembly. Blue: complete and single-copy genes; orange: complete and duplicated genes; grey: fragmented genes; yellow: missing genes.

The use of the BUSCO software facilitated an overview of the completeness of the assemblies obtained in this work. In BUSCO, the *embryophyta_odb9* orthologous database for Magnoliophyta plants (flowering plants) has a total of 1,440 BUSCO orthologs groups whose completeness will depend on the assembly of holm oak. According to BUSCO analysis, the RAY hybrid assemblies generated the most complete transcriptomes with 1,116 and 1,057 complete sequences of which 1,085 and 1,036 were single copy sequences in holm oak, respectively (Fig 2). From Illumina reads, MIRA (1,031) generated a more complete transcriptome than RAY (807 bp) and TRINITY (66) (Fig 2). From Ion Torrent reads, TRINITY generated the most complete transcriptome (779), followed by MIRA (436) and RAY (411) (Fig 2).

### Annotation of the best *Q*. *ilex* transcriptome from each sequencing platform

The annotation was performed by the Sma3s v2 algorithm. It is worth mentioning that Blast2GO, rather than Sma3s v2, was previously used in [17]. However, the annotation increased from 31,972 total transcripts annotated by Blast2GO to 62,628 total transcripts recently annotated by Sma3s v2 using the MIRA assembly [18], both from Illumina reads. From Ion Torrent reads, 74,058 total transcripts were annotated by the TRINITY assembly while from the hybrid transcriptome assembly, 34,360 transcripts were annotated using the RAY assembly. Regarding the partial hybrid transcriptome, around 33,694 transcripts were annotated using the same assembly as for the hybrid transcriptome.

In order to facilitate the access and use of the *Q*. *ilex* transcriptome sequencing data, the raw data in the FASTQ format was deposited in the Sequence Read Archive (SRA-NCBI) database with accession numbers: SRR7456533 and SRR7454228 (Ion Torrent sequencing platform using 10 ng and 50 ng of total RNA, respectively) and SRR5815058 (Illumina sequencing platform), and the whole transcriptome was uploaded to the holm oak database (http://www.uco.es/probiveag/holm-oak-database.html; section “data”).

### Gene ontology classification of *Quercus ilex* transcripts

**Fig 3:**
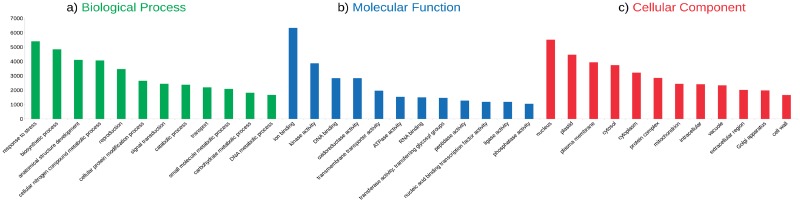
Histogram of GO classification of assembled *Quercus ilex* transcripts. Horizontal bar charts of the distribution of GO associated with the holm oak transcripts represented in the three main GO categories: biological processes (**a**), molecular functions (**b**) and cellular components (**c**). The first twelve transcripts assigned to each GO category are shown and the remaining transcripts assigned to each GO category are shown in S1 Table.

Gene ontology (GO) for the *Q*. *ilex* transcripts obtained from the hybrid assembly were analyzed by Sma3s v2 to classify the functions of the assembled transcripts in terms of biological process, molecular function and cellular component (Fig 3; S1 Table). Within the biological processes, more transcripts were assigned to response to stress and biosynthetic processes, followed by anatomical structure development and cellular nitrogen compound metabolic processes (Fig 3a; S1 Table). In the case of the molecular functions, many transcripts were associated with ion binding, kinase activity, oxidoreductase activity and DNA binding (Fig 3b; S1 Table). Finally, in the cellular component category, the transcripts were mainly classified in terms of nucleus, plastid and plasma membrane (Fig 3c; S1 Table). A high number of transcripts (5,405 transcripts) of holm oak were assigned to response to stress (Fig 3a; S1 Table), of which 46 (0.85%) transcripts were directly included in the drought stress category, according to our annotation (S2 Table). Some of the transcripts related to drought stress were: UDP-Glucosyltransferase; TCTP (Translationally Controlled Tumor Protein); NACs (82-77-53-46) transcription factors; DICP (Drought Inducible Cysteine Proteinase); PUF (Pumilio/Fem-3-binding factor), APUM (*Arabidopsis* Pumilio RNA binding protein) and PUM (Pumilio) RNA-binding proteins; PXG4 (Peroxygenase 4); PAL and PAL5 (Phenylalanine Ammonia Lyase); NH2 and NH8 (Nam Line Protein); DRS1 (Drought Sensitive 1 protein); Drought-induced protein RDI; At3g62550-drought responsive ATP-binding motif containing protein; UGT7G3 Anthocyanidin 3-O-glucosyltransferase 2; and TCM_034302 (Chloroplastic drought-induced stress protein) (S2 Table).

On the other hand, we also considered a representative sample of 2,000 random transcripts to be classified by GO terms (biological process, molecular function and cellular component) (S3 Table). Within the biological processes, more transcripts were assigned to response to stress, biosynthetic processes and anatomical structure development in all the transcriptome assemblies built in this study (S3 Table). Within the molecular functions, the majority of transcripts in all the assemblies were grouped into ion binding, DNA binding and kinase activity (Fig 3b; S1 Table). Finally, in the cellular component category, the transcripts were mainly classified in terms of nucleus, plastid and cytosol (Fig 3c; S1 Table).

In addition to the GO classification, the *Q*. *ilex* transcripts were classified in terms of biological process, pathway and cellular component at the Universal Protein Resource (UniProt). Within the biological processes, more transcripts were assigned to plant defense, followed by transport and transcription (S1 Table). Within the pathway category, the majority of transcripts were associated with the response to stress and biosynthetic processes (S1 Table). Finally, in the cellular component category, the transcripts were mainly classified in terms of the membrane and nucleus (S1 Table).

To further understand the degree of transcript overlap between each of the assemblers-platforms, we created a matrix in which each cell represents the overlap between two assemblers-platforms used in this study (Table 2). The highest percentage overlap was observed when TRINITY-Ion Torrent was blasted with MIRA-Ion Torrent (96.58%), followed by MIRA-Illumina blasted with MIRA-Ion Torrent (95.02%) and RAY-Ion Torrent blasted with hybrid assembly (90.36%) (Table 2). As expected, the lowest percentage overlaps were observed when all the assemblies were blasted with TRINITY-Ion Torrent, obtaining the lowest overlap between MIRA-Ion Torrent and TRINITY-Illumina (19.51%) (Table 2).

**Table 2:** Blast percentage matrix of all the transcriptomes built in holm oak. Each cell in the matrix represents the overlap between two assemblers-platforms.

	**RAY-Hybrid**	**RAY-Partial hybrid**	**MIRA-Illumina**	**MIRA-Ion Torrent**	**RAY-Illumina**	**RAY-Ion Torrent**	**TRINITY-Illumina**	**TRINITY-Ion Torrent**
**RAY-Hybrid**	**99,94**	61,83	50,03	67,25	56,52	54,28	46,89	64,48
**RAY-Partial hybrid**	86,95	**99,95**	63,20	78,65	63,49	60,83	48,48	76,55
**MIRA-Illumina**	89,96	86,80	**99,94**	95,02	83,84	72,02	34,45	94,79
**MIRA-Ion Torrent**	71,85	68,62	74,22	**99,97**	61,34	55,52	19,51	84,27
**RAY-Illumina**	88,86	72,68	76,16	74,76	**99,92**	52,24	50,64	75,33
**RAY-Ion Torrent**	90,36	77,93	64,26	88,56	61,27	**99,98**	36,24	85,30
**TRINITY-Illumina**	73,59	57,55	54,25	68,58	59,57	43,06	**99,98**	66,55
**TRINITY-Ion Torrent**	87,15	82,97	86,85	96,58	77,23	73,52	40,70	**100,00**

### Protein annotation in holm oak

The protein identification carried out with Proteome Discoverer 2.1 by using a translated version of *Q*. *ilex* transcriptome assemblies gave a successful result (Table 3). In terms of total number of proteins from the Illumina translated transcriptome, 1,878, 1,930 and 565 proteins were identified after using the MIRA, RAY and TRINITY assemblers, respectively, while from the Ion Torrent translated transcriptome, 2,242, 2,356 and 2,395 proteins were identified after using the MIRA, RAY and TRINITY assemblers, respectively. Both hybrid and the partial hybrid assemblies to obtain the holm oak proteome were also carried out in this work, giving rise to a total of 1,899 and 1,801 proteins after using the RAY assembler, respectively (Table 3).

**Table 3:** Summary of the total number of proteins annotated in holm oak.

Protein identification								
	Illumina			Ion Torrent			Hybrid	Partial hybrid
	MIRA	RAY	TRINITY	MIRA	RAY	TRINITY	RAY	
Total	1878	1930	565	2242	2356	2395	1899	1801
Mean length	440,12	277,42	130,14	136,91	164,69	242,00	321,53	351,77
Annotated	1818 (97%)	1881 (97%)	547 (97%)	1972 (88%)	2303 (98%)	2373 (99%)	1841 (97%)	1753 (97%)
Unique genes	1492 (82%)	1508 (80%)	460 (84%)	1365 (69%)	1523 (66%)	1284 (54%)	1522 (83%)	1492 (85%)
With at least 1 unique peptide	1878 (100%)	1930 (100%)	565 (100%)	2242 (100%)	2356 (100%)	2395 (100%)	1899 (100%)	1801 (100%)
With at least 2 unique peptides	995 (53%)	1111 (58%)	257 (45%)	681 (30%)	1153 (49%)	1258 (53%)	1128 (59%)	1100 (61%)
With at least 3 unique peptides	620 (33%)	762 (39%)	133 (24%)	244 (11%)	629 (27%)	776 (32%)	795 (42%)	804 (45%)
With at least 7 unique peptides	172 (9%)	192 (10%)	18 (3%)	17 (1%)	67 (3%)	159 (6%)	212 (11%)	251 (14%)

The total number of annotated proteins was quite similar to the data described in the total number of proteins identified from each translated transcriptome (Table 3). A total of 1,818 (97%) (MIRA), 1,881 (97%) (RAY) and 547 (97%) (TRINITY) annotated proteins were identified from the Illumina translated transcriptome, while a total of 1,972 (88%) (MIRA), 2,303 (98%) (RAY) and 2,373 (99%) (TRINITY) annotated proteins were identified from the Ion Torrents translated transcriptome (Table 3). The highest number of unique genes (or unique translated protein sequences) was identified in TRINITY-Illumina (84%) and RAY-hybrid (83%) (Table 3). The hybrid assembly showed 1,899 proteins, of which 1,841 (97%) were annotated proteins and 1,522 unique genes (83%), and the partial hybrid assembly showed 1,801 proteins, of which 1,753 (97%) were annotated proteins and 1,492 unique genes (85%) (Table 3).

## Discussion

In the present work, we evaluate several procedures to build an accurate *de novo* transcriptome for *Q*. *ilex* from a mixture of experimental raw sequence read data and statistical approaches. An accurate holm oak transcriptome has already been described by this research group [17], and therefore this present study is now focused on a comparative analysis of two sequencing platforms, Illumina and Ion Torrent, and three different assemblers (TRINITY, MIRA and RAY) used to assemble all the clean raw data obtained in the holm oak transcriptome analysis. Moreover, a *de novo* hybrid transcriptome using both sequencing platforms was built and compared to the transcriptomes obtained through Illumina and Ion Torrent alone. The *de novo* hybrid transcriptome was only assembled using RAY, as mentioned above, as neither the TRINITY nor MIRA assemblers are recommended for the assembly of a hybrid transcriptome using such a large amount of sequences. A *de novo* hybrid assembly is a setting up process of sequences by using two or more sequencing platform data. This kind of assembly was developed due to the limitations of each sequencing platform. The Illumina technology produces low percentage substitution errors (0.3–3.8%) [37–38], and the Ion Torrent technology presents indels (insertion/deletion error types) at a raw rate of 2.84% [39]. By using a hybrid assembly algorithm, we attempted to correct those errors generated in both technologies. This strategy is currently used to correct the elevated rate of errors in third generation sequencing reads [40], using high quality short reads from second generation sequencing platforms. Moreover, the use of a partial hybrid transcriptome helped in the estimation of the good quality of the hybrid transcriptome, due mainly to the correction of errors commented above rather than the read depth of using both sequencing platforms. Guerrero-Sanchez et al. [17] previously annotated 31,972 total transcripts by Blast2GO from Illumina reads assembled by MIRA, which increased the genetic information available at that time in the databases of holm oak (659 sequences on nucleotide database and 88 EST databases annotated by NCBI, (https://www.ncbi.nlm.nih.gov/)). The genetic information of holm oak was increased to 62,628 total transcripts annotated using Sma3s v2, rather than Blast2GO [18], from Illumina reads assembled by MIRA. Additionally, 74,058 and 34,360 total transcripts were obtained in this work using Sma3s v2 from Ion Torrent reads assembled by TRINITY and the hybrid transcriptome assembled by RAY, respectively.

Both sequencing platforms and the assemblers available should be considered carefully, when looking for the best option, especially when there is scarce information about the species under study, as in holm oak. Bradnam et al. [16] reported in the Assemblathon 2 context that more than a single assembly or a single metric should be carried out to assess the quality of an assembly. This is due to the read lengths, read counts and error profiles that are produced by different NGS technologies [16]. So, we compared the *de novo* holm oak Illumina transcriptome previously described by [18] to the *de novo* Ion Torrent transcriptome and *de novo* hybrid transcriptome, with the aim of building a more complete *de novo* holm oak transcriptome. Moreover, the efficiency in the use of computational resources should be considered in a transcriptome analysis. The assembler should be chosen according to the computational resources required to process the clean raw data, since the computer resources needed represent a clear limitation for performing the assembly. In this study, the RAY assembler proved more convenient in all the transcriptomes built due to the efficient use of computational resources (S2 Fig). Regarding the assembly structure, the TRINITY-Ion Torrent assembly annotated a higher number of sequences, while the MIRA-Ion Torrent assembly shared more sequences with *Q*. *robur* and *Q*. *petrea* transcriptomes (Fig 1).

With regard to completeness assessment, the hybrid transcriptome yielded the most complete sequences in relation to the ortholog alignment, followed by MIRA-Illumina and TRINITY-Ion Torrent assemblies (Fig 2). The Ion Torrent assemblies contain more duplicated and fragmented sequences than Illumina and hybrid assemblies (Fig 2), which may be due to both the structure of the reads and, single-end in Ion Torrent and paired-end in Illumina. Despite these differences, the Ion Torrent technology gave better assembly structure and protein identification, in addition to being quicker and cheaper than the paired-end sequencing commonly used in the Illumina platform [19]. On the other hand, the hybrid transcriptome was used to carry out the GO ontology classification as this transcriptome built the most complete sequence in relation to the ortholog alignment (Fig 2), identifying the highest number of unique peptides with more than 3 (Table 2) and being the most efficient in the use of resources during the assembly (S2 Fig).

It was remarkable that the higher number of transcripts observed in the GO biological processes was related to the stress response (46 out of 5,405; 0.85%). Conversely, *Q*. *robur* did not show any stress response related transcripts [29], while they have been observed for other related species such as *Castanea dentata* and *Eucalyptus grandis* [29]. The *Q*. *ilex* transcriptome annotations revealed interesting information about its biology, which can be used in a genetic study devoted to investigating one of the major problems that threaten this species, drought [41]. A previous study has assessed the effect of the drought in holm oak by a proteomic analysis, reporting a large list of proteins whose levels changed under drought conditions [42]. Interestingly, in this study, an overview of drought-resistant genes in holm oak is provided from a transcriptomic approach. Although the number of transcripts related to drought stress identified in this work was lower than the number of proteins identified previously [42], those transcripts are directly related to drought rather than to general stress response. Nevertheless, all the proteins identified by Simova-Stoilova et al. [42] were also identified in our annotations but some of them were not included in the drought stress classification.

Regarding the identification of proteins by Proteome Discovered 2.1, RAY translated assembly from Illumina reads identified more proteins than TRINITY and MIRA, and from Ion Torrent reads, the three assemblers used in this study identified similar numbers of proteins (Table 3). However, as a general tendency, all the Ion Torrent translated assemblies showed more proteins than the Illumina assemblies. The hybrid assemblies showed quite similar number of proteins as the Illumina translated assemblies.

## Conclusions

**Fig 4:**
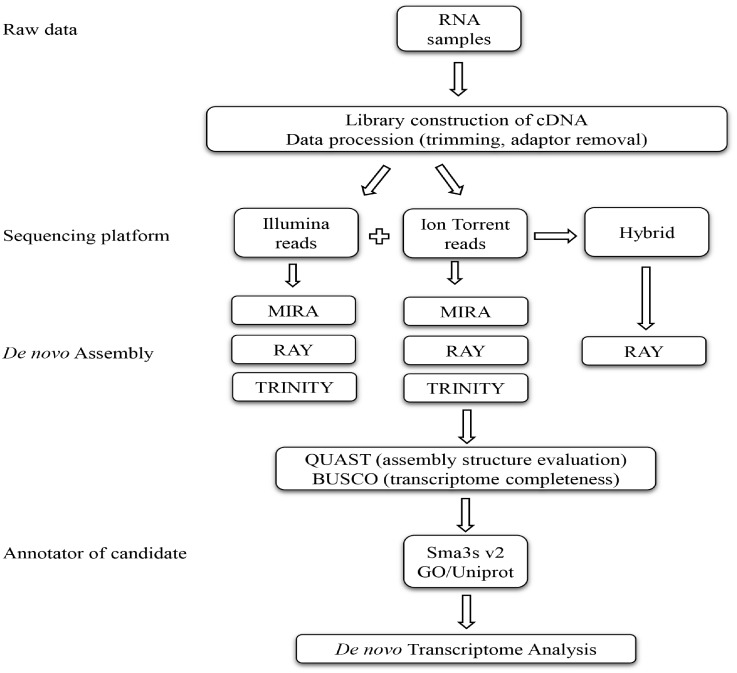
Experimental work flow showing the steps carried out and bioinformatic utilities used for a transcriptome analysis.

To obtain genetic information in a non-model species, such as holm oak whose genome has not been yet sequenced, remains a challenge. The comparison between Illumina and Ion Torrent sequencing platforms using different assemblers was carried out to further our knowledge of the *de novo* holm oak transcriptome previously described [17–18]. We found that an increase of genetic information could be obtained when the Ion Torrent transcriptome and the hybrid (Illumina and Ion Torrent together) transcriptome were used. This work sheds light on *Q*. *ilex* biology. Besides, the optimized workflow described here for the holm oak transcriptome will help to progress on other non-model species (Fig 4). The annotated transcripts and proteins could be used to carry out differential expression studies of different biotic and abiotic stresses such as drought or resistance to *Phytophthora cinnamomi*, which seriously affect the biology of holm oak, and as a tool of validation for the whole genome sequencing of holm oak.

## Supporting information

S1 Table Total number of transcripts included in the GO and Uniprot classification in holm oak. (XLSX)

S2 Table List of transcripts related to drought stress in the holm oak transcriptome. (XLSX)

S3 Table A representative sample of 2,000 transcripts grouped into the GO classification in holm oak. (XLSX)

S1 Fig Distribution of sequence lengths over all sequences used in this study. (EPS)

S2 Fig Efficiency in the use of computational resources in each assembler used in this study (RAY, MIRA and TRINITY) from Illumina clean raw data. Ncpus indicates how many central processing units (CPUs) are used by the software, Ncpus_sys indicates how many CPUs are used by the system, Mem indicates RAM memory and Process_creation indicates how many files are created.
(EPS)

S3 Fig Efficiency in the use of computational resources in each assembler used in this study (RAY, MIRA and TRINITY) from Ion Torrent clean raw data. Ncpus indicates how many central processing units (CPUs) are used by the software, Ncpus_sys indicates how many CPUs are used by the system, Mem indicates RAM memory and Process_creation indicates how many files are created.
(EPS)

S4 Fig Efficiency in the use of computational resources in the RAY assembler from hybrid transcriptome (a) and partial hybrid transcriptome clean raw data (b). Ncpus indicates how many central processing units (CPUs) are used by the software, Ncpus_sys indicates how many CPUs are used by the system, Mem indicates RAM memory and Process_creation indicates how many files are created.
(EPS)
